# Bioinformatics and Moonlighting Proteins

**DOI:** 10.3389/fbioe.2015.00090

**Published:** 2015-06-24

**Authors:** Sergio Hernández, Luís Franco, Alejandra Calvo, Gabriela Ferragut, Antoni Hermoso, Isaac Amela, Antonio Gómez, Enrique Querol, Juan Cedano

**Affiliations:** ^1^Institut de Biotecnologia i Biomedicina and Departament de Bioquímica i Biologia Molecular, Universitat Autònoma de Barcelona, Barcelona, Spain; ^2^Laboratorio de Inmunología, Universidad de la República Regional Norte-Salto, Salto, Uruguay; ^3^Cancer Epigenetics and Biology Program, Institut d’Investigació Biomèdica de Bellvitge, L’Hospitalet de Llobregat, Barcelona, Spain

**Keywords:** multitasking, multifunctional, moonlighting protein, bioinformatics, protein function, protein evolution

## Abstract

Multitasking or moonlighting is the capability of some proteins to execute two or more biochemical functions. Usually, moonlighting proteins are experimentally revealed by serendipity. For this reason, it would be helpful that Bioinformatics could predict this multifunctionality, especially because of the large amounts of sequences from genome projects. In the present work, we analyze and describe several approaches that use sequences, structures, interactomics, and current bioinformatics algorithms and programs to try to overcome this problem. Among these approaches are (a) remote homology searches using Psi-Blast, (b) detection of functional motifs and domains, (c) analysis of data from protein–protein interaction databases (PPIs), (d) match the query protein sequence to 3D databases (i.e., algorithms as PISITE), and (e) mutation correlation analysis between amino acids by algorithms as MISTIC. Programs designed to identify functional motif/domains detect mainly the canonical function but usually fail in the detection of the moonlighting one, Pfam and ProDom being the best methods. Remote homology search by Psi-Blast combined with data from interactomics databases (PPIs) has the best performance. Structural information and mutation correlation analysis can help us to map the functional sites. Mutation correlation analysis can only be used in very specific situations – it requires the existence of multialigned family protein sequences – but can suggest how the evolutionary process of second function acquisition took place. The multitasking protein database MultitaskProtDB (http://wallace.uab.es/multitask/), previously published by our group, has been used as a benchmark for the all of the analyses.

## Introduction

Multitasking and moonlighting proteins refer to those proteins with two or more functions performed by a single polypeptide chain. They were initially reported by Wistow and Piatigorsky in the late 1980s when structural *lens crystallins* turned out to be identical to previously known metabolic enzymes (Wistow and Piatigorsky, 1987). Piatigorsky proposed *gene sharing* for these proteins (Piatigorsky, 2008). The term *moonlighting* was coined by Jeffery (1999), who intended a more restrictive definition, as this term does not encompass the cases of gene fusions. Moonlighting proteins present alternative functions which are mostly affected by cellular localization, cell type, oligomeric state, concentration of cellular ligands, substrates, cofactors, products, or post-translational modifications (Wool, 1996; Jeffery, 1999, 2003, 2004, 2009, 2014; Gancedo and Flores, 2008; Piatigorsky, 2008; Nobeli et al., 2009; Huberts and Van Der Klei, 2010; Henderson and Martin, 2011; Copley, 2012). In many cases, a protein uses a combination of these mechanisms to switch between functions. As stated by Jeffery, current moonlighting proteins “appear to be only the tip of the iceberg.” Tompa et al. (2005) have reported that some moonlighting proteins might correspond to the class of proteins named intrinsically disordered proteins (IDP). However, using IDP prediction programs, we have shown that they seem to be in the same percentage as other proteins (Hernández et al., 2012). Although some findings suggest involvement of a protein in extra functions, i.e., they can be found in different cellular localizations or in amounts exceeding those required for their canonical function, usually moonlighting proteins are experimentally revealed by serendipity. Therefore, it would be helpful if Bioinformatics could predict multifunctionality, especially because of the large amounts of sequences coming from genome projects (Hernandez et al., 2014a). During the development of our previous work aimed at trying to find bioinformatics approaches to predict multitasking proteins, we encountered the difficulty of collecting enough examples of such proteins by the lack of a broad database; thus, we have collected and compiled a database of multifunctional proteins (Hernandez et al., 2014b), which is accessible at http://wallace.uab.es/multitask/. It shows about 300 multitasking proteins plus 350 additional examples not confirmed yet. In addition, another database devoted to the true moonlighting proteins, MoonProt, from Jeffery’s group is at www.moonlightingproteins.org (Mani et al., 2015).

In a previous work, we have explored the possibility of identifying moonlighting proteins by using bioinformatics approaches (Gomez et al., 2003) and protein interactomics database (PPI) information (Gomez et al., 2011), or based on whether they belong to the intrinsically disordered protein class (IDP) (Hernández et al., 2012). Another approach used to identify moonlighting proteins has also been reported (Khan et al., 2012, 2014; Khan and Kihara, 2014). They use several approaches, such as protein–protein interaction (PPI) databases, gene expression, phylogenetic profile, and genetic interaction networks, which they can cluster GO annotations of moonlighting proteins into multiple groups reflecting their diverse functions and identifying 33 novel moonlighting proteins in *Escherichia coli*. PPI databases should contain information on moonlighting proteins and provide suggestions for further analysis in order to prove their multifunctionality. It is generally considered that experimental data from proteomics contain many false positives, estimated to be up to about 20% (Prieto et al., 2006; Lievens et al., 2010). This may easily induce proteomics researchers to consider most of the unexpected partners as false positives. This may represent a handicap for identifying true multifunctional proteins. For example, ribosomal proteins are generally considered as false positives in the yeast two-hybrid method. However, this kind of proteins is prone to be moonlighting and a number of them could be true positives (Wool, 1996). Another approach using PPI databases is that of Brun’s group (Becker et al., 2012), which from human interactome networks and Pfam domains can predict multiclustered proteins as moonlighting candidates.

In the present work, using our database as a benchmark, we analyze and describe several approaches that use sequences, structures, interactomics, and current bioinformatics algorithms and programs to try to overcome this problem. Among them are (a) remote homology searches using Psi-Blast, (b) detection of functional motifs and domains, (c) analysis of data from PPI databases, (d) match the query protein sequence to 3D databases (i.e., algorithms such as Pisite), and (e) mutation correlation analysis between amino acids by algorithms such as Mistic. Programs designed to identify functional motifs and domains detect mainly the canonical function but usually fail in the detection of the moonlighting one, with Pfam and ProDom being the best ones. Remote homology search by Psi-Blast combined with data from interactomics databases (PPIs) has the best performance. Structural information and mutation correlation analysis can help us to map the functional sites. Mutation correlation analysis can be used only in very restrictive situations; it requires the existence of a multialigned family of protein sequences, but can suggest how the evolutionary process of the second function acquisition took place.

However, although bioinformatics analyses can help to suggest which proteins are multifunctional, identifying true positives must be demonstrated experimentally.

## Materials and Methods

### Databases

The database of multifunctional proteins, MultitaskProtDB, (Hernandez et al., 2014b) is accessible at the web page http://wallace.uab.es/multitask/. Another database of moonlighting proteins is MoonProt (Mani et al., 2015), accessible at http://www.moonlightingproteins.org. However, all the bioinformatics analyses performed in the present work have been run using the protein set from MultitaskProtDB.

Protein–protein interaction partners for moonlighting proteins have been checked in the APID server (Prieto et al., 2006) at http://bioinfow.dep.usal.es/apid/index.htm. APID comprises most of the proteomics data reported at MINT, DIP, BioGRID, IntAct, HPRD, and BIND. In addition, it performs directly a GO screening^1^ (Ashburner et al., 2000). We have considered that the proteomics data predict the second function of a moonlighting protein if the PPI database identifies a *Molecular Function* or, in some cases, a *Biological Process* according to the Gene Ontology annotation (see text footnote 1), which is in agreement with the expected additional function. In order to filter hits and to improve the accuracy, it is advisable to perform a Gene Ontology enrichment analysis using the GOStat R package (Beissbarth and Speed, 2004) as previously reported (Gomez et al., 2011).

### Protein sequence analyses

Remote homology analysis on the NCBI non-redundant database was done using Psi-Blast (Altschul et al., 1997), accessible at http://www.ncbi.nlm.nih.gov/BLAST. The search was performed with the following settings and with a maximum of five iterations with default parameters (*Filter*: “F,” *gap_extend*: “1,” *expect*: 10, and *gap_open*: 11). Another problem is that since Psi-Blast output arranges the hits according to their mathematical scores, instead of their biological ones, the true hit cannot be found in the top positions, but in the lower ones. Therefore, the Psi-Blast output list has been rearranged by means of the ByPass fuzzy logic program: http://bypass.uab.cat/wiki/ (Gomez et al., 2008). ByPass uses fuzzy logic to rearrange the Blast or Psi-Blast output and moves up to top-position, as the putative true positives, proteins, where the function can be identified. Bypass was performed with default parameters (4, 0, 0, 5, 1) and, from the last iteration (the fifth or a previous one if converged before), up to 100 hits were retrieved with *E*-value scores better than 0.01.

Motif and domain screening was performed using InterPro (Hunter et al., 2012) accessible at http://www.ebi.ac.uk/Tools/pfa/iprscan/. A database of the protein motifs alignment Blocks (Henikoff et al., 1999) was also used, accessible at http://blocks.fhcrc.org. In theory, it is more advisable to use InterPro, because Blocks has not been updated since 2006. Another program, Pfam (Finn et al., 2006), also supported by InterPro, allows the user to perform a search in a more or less restricted set of domains (PfamA and PfamB, respectively). PfamB depicts a less restricted output containing less specific matches and so can be better at identifying putative moonlighting domains. Nevertheless, and by default, Pfam at InterPro only uses the PfamA output. PfamB has to be activated by the user at http://pfam.xfam.org/search.

Prediction of the cellular sublocalization was done by two programs that display in their outputs different localizations ordered according to their respective scores. They are Psort (Nakai and Horton, 1999) at http://www.psort.org/ and ProtLoc (Cedano et al., 1997) at http://bioinf.uab.es/cgi-bin/trsdb/protloc.cgi. Ideally, the two top hits should be the ones related to the localizations of the pair of moonlighting functions.

### Mapping the structural/functional sites on the protein sequence using homology to 3D structures

Programs to identify functional sites can help to disclose additional functions of the protein if its 3D structure is known (Aloy et al., 2001). To check if the main structural/functional sites for both functions can be disclosed from the protein sequence, we have used Pisite (Higurashi et al., 2009), a program for mapping functional domains. This program is available at the web http://pisite.hgc.jp/. Pisite is a web database of protein interaction sites that works by aligning the query protein sequence to those present in the PDB. Phyre (Kelley and Sternberg, 2009) is another program for structure prediction and modeling that we have used, available at http://www.imperial.ac.uk/phyre/. The program also discloses key functional amino acid residues, which can help to identify additional functional sites.

### Mutation correlation analyses

Coevolution amino acidic analyses were done using Mistic (Mutual Information Server To Infer Coevolution) server http://mistic.leloir.org.ar (Simonetti et al., 2013). Mistic is a web server providing a graphical representation of the information contained in a multialignment of sequences. This program enables estimating the coevolutionary relationship between two amino acid positions in a protein family from the positional correlations. In this way, the user can identify structurally or functionally relevant amino acid positions (Hernandez et al., 2014a).

## Results

All the following results refer to the analysis of the multitasking proteins contained in the MultitaskProtDB database. The different functions have been labeled as “canonical” or “moonlighting,” in the database and in the present work, but this has no biological relevance and merely refers to the historical order of the discovery of the biological function. There are different ways to assign a function to a protein sequence of unknown function, but the most used methods do the functional annotation by the application of the transitivity property. If a protein of unknown function has a certain degree of similarity with an annotated one, then it is assumed that they share the same function. But, if we have a high degree of redundant information (i.e., a large set of related sequences), this redundancy can be used to infer which function it is by means of pattern extraction. In this case, we can use the extracted pattern to infer the function instead of using the individual sequences. The extracted pattern can also be used to identify the key amino acids for their function. Another way to disclose the important amino acids in a protein needed for its function possible, especially when it is involved in the interaction with a complex, is by means of the simulation of the molecules at the three-dimensional level. All these systems could be used to predict how exactly the amino acids are implicated in this function. But this method has the limitation that protein structures are required for each tested molecule and, moreover, high-computational requirements in terms of computational power and time are needed. All these classical strategies or their combinations can be used to infer function also in moonlighting proteins.

### Homology/remote homology searches

**Figure 1:**
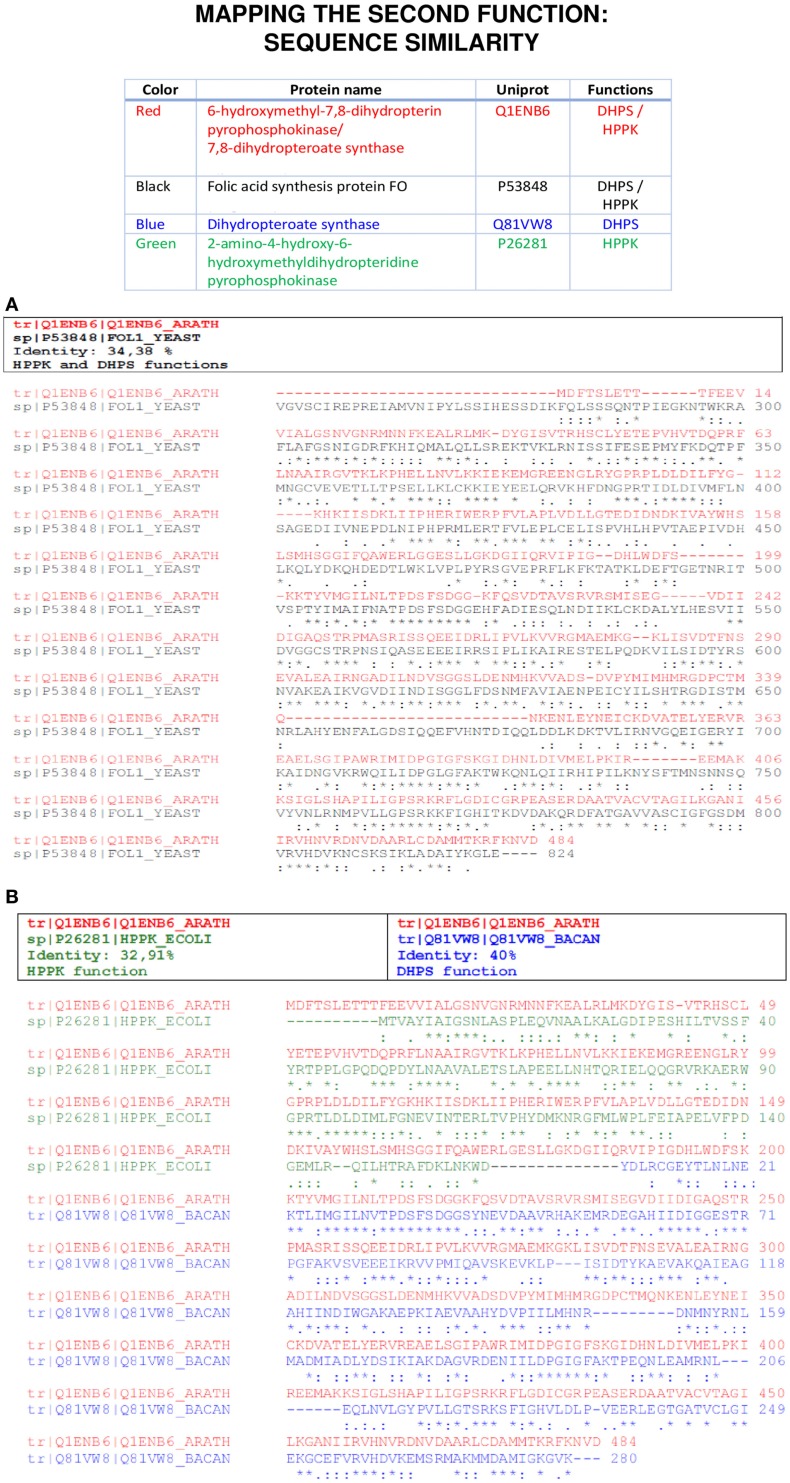
**An example of second function mapping using sequence approaches**. In **(A)**, a moonlighting protein sequence (red) is aligned with ClustalW to another moonlighting protein sequence of a different organism (black) that was found after a BLASTP analysis. In **(B)**, the same approach is used and the moonlighting protein sequence (red) is aligned with two monofunctional proteins (green/blue), each one in a region of the moonlighting protein; therefore, mapping the canonical and moonlighting functions of the original protein.

Blast, and especially Psi-Blast, can detect a number of multitasking proteins indicated as those having more than one stretch aligned to different targets. For instance, Figure 1 shows an example for the protein 2-amino-4-hydroxy-6-hydroxymethyldihydropterine pyrophosphokinase (HPPK) and dihydropteroate synthase (DHPS). This example can also represent a case for gene fusions leading to a multitasking protein.

The remote homology algorithm Psi-Blast is especially suitable to disclose moonlighting proteins because it can identify stretches of conserved amino acid residues from different domains (Gomez et al., 2003; Khan et al., 2012). As in PPI database searches (see next section), the output depicts a large list of hits and the researcher does not know *a priori*, which of them will be true positives, and it is the careful analysis of the different predictions and the experimental data that can suggest a true hit. The functional annotation was inspected in order to check whether the matching entries contained canonical and moonlighting annotations. A classical problem with homology programs is due to the large number of sequences in protein families, for example, ribosomal proteins, which are typical moonlighting proteins that collapse the output causing the putative second function to remain hidden. The non-redundant database allows running Psi-Blast while collapsing all the GenBank entries that share the same sequence, but it is also possible to search for homologous sequences in manually curated databases like SWISS-PROT or any other set of sequences. As described in Section “Materials and Methods,” ByPass can also help to disclose the true hits, since we have found that among the sequences “moved-up” with improved scores by ByPass, there are hits corresponding to moonlighting functions; however, in most cases, ByPass does not shift them exactly in the first and second hit positions. We checked whether the ByPass algorithm moves both the canonical and moonlighting function to top positions. But not always does it, so this type of method does not help us to solve the problem of having to analyze long lists of items provided by Psi-Blast. There are examples, such as enolases, in which the moonlighting adaptation may be a process more common than expected and often just implies redesigning a small portion of the protein sequence. Therefore, a number of proteins presenting very similar sequences may be prone to present the same moonlighting function and the rest not. Therefore, most programs like ByPass, which make a global calculation of similarity between the amino acid sequences of the proteins analyzed, may fail in detecting local changes in them. In conclusion, following the type of criteria used by ByPass, we cannot guarantee that the enrichment at the top positions in the output would correspond to true-positive cases of moonlighting functions.

We have considered as positive Psi-Blast matches those describing the function in a broad sense in any position of the output. For cases in which the moonlighting protein is an enzyme and a transcription factor (the most abundant pair reported in MultitaskProtDB), we can consider as a predictor that the second hit is predicted as a *transcription factor* or even a *Zn Finger domain*. Column 4 of Table 1 shows some examples of moonlighting proteins identified by remote homology. From the 288 moonlighting proteins in the MultitaskProtDB database, Psi-Blast identifies the second (moonlighting) function in about half of the cases when considering a positive hit in a more broad sense than in the strict GO annotation, because annotations in sequence databases, such as NCBI, are quite loose and vague. It could be expected that the specificity of moonlighting identification would be improved running Psi-Blast against the GO annotation. Although the GO annotation compacts the hits to few functional categories, reducing therefore the effort when analyzing a large amount of entries, so we have found that the sensitivity in detecting some moonlighting functions is lowered. However, current GO annotations contain only about 9500 molecular functions; so, many functional annotations from sequence databases cannot be found at GO.

**Table 1:** **Examples of moonlighting proteins prediction combining PPI databases and Bypass**.

Canonical function	Moonlighting function	PPI partners (only some hits are shown)	Bypass output (only some hits are shown)
Phosphoglucose isomerase	Neurotrophic factor Neuroleukin Autocrine motility factor Nerve growth factor	GO:4842 autocrine motility factor receptor 2 GO: 31994 insulin-like growth factor binding protein 3	giI17380385 Glucose 6 phosphate isomerase Autocrine motility factor Neuroleukin
Pyruvate kinase	Tyroid hormone-binding protein	GO:3707 nucelar hormone receptor member nhr-111 GO: 9914 sex hormone binding globulin GO: 5179 atrial natriuretic factor	giI20178296 Pyruvate kinase isozymes Cytosolic thyroid hormone-binding protein
Ribosomal protein S3 (human)	Apurinic/apirymidinic endonuclease	GO: 31571 DNA damage binding protein 1 GO: 3735 S27 ribosomal protein	giI290275 Ribosomal protein S3 AP endonuclease DNA repair
Ure2	Glutathione peroxidase	GO: 6808 nitrogen regulatory protein	giI173152; gi449015276 Glutathione transferase-like protein Nitrogen catabolite repression transcriptional regulator
P0 ribosomal protein	DNA repair	GO: 6281, FACT complex subunit SSRP1	
Vhs3-phosphopantothenoylcysteine decarboxylase subunit Vhs3	Regulator of serine/threonine protein phosphatase	GO: 4724, serine/threonine-protein phosphatase PP-Z1	gi|254572327|ref|XP_002493273.1|Negative regulatory subunit of the protein phosphatase 1 Ppz1p
Epsin	Organizing mitotic membranes/influencing spindle assembly	GO: 7067, cell division control protein 2 homolog	gi|2072301|gb|AAC60123.1|mitotic phosphoprotein 90
Alpha-crystallin A chain	Heat-shock protein	GO: 6986, Heat shock protein beta-1	gi|1706112|sp|P02489.2|CRYAA_HUMAN RecName: Full = Alpha-crystallin A chain; AltName: Full = Heat shock protein beta-4
Hexokinase	Transcriptional regulation	GO: 16563, metallothionein expression activator	gi|254573908|ref|XP_002494063.1|Non-essential protein of unknown function required for transcriptional induction
Ribosomal protein L7	Autogenous regulation of translation	GO: 6414, 60S ribosomal protein L7a	gi|339256006|ref|XP_003370746.1|eukaryotic translation initiation factor 2C 2
PIAS1 (E3 SUMO-protein ligase PIAS1)	Activation of p53	GO: 7569, cellular tumor antigen p53	gi|58176991|pdb|1V66|A Chain A, solution structure of human P53 binding domain of Pias-1

Finally, it deserves to be mentioned that only 8% of the moonlighting proteins of the database are identified by Psi-Blast and InterPro at the same time (see “Sequence Searches Using Motif/Domain Programs” below).

### Interactomics database searches

We have proposed previously that protein–protein interaction (PPI) databases combined with sequence similarity analysis can help to predict protein function (Espadaler et al., 2008) and that PPI databases should also contain information on moonlighting proteins and provide suggestions for further analyses in order to prove their multitasking properties (Gomez et al., 2011). Interactomics partners of a protein could suggest the function or functions of a protein (“guilty-by-association”), at least at the level of GO’s biological process. We considered that interactomics databases correctly disclose a second function for the moonlighting protein if the PPI database identifies a *Molecular Function* or, in some cases, a *Biological Process* according to the Gene Ontology annotation, which is in agreement with the additional function described in our database. Then, in order to filter hits and improve the accuracy, it is advisable to perform a Gene Ontology enrichment analysis. For each moonlighting protein included in APID, we collected the GO terms of the interaction partners and computed GO term enrichment using the GOStat R package (Beissbarth and Speed, 2004). This function will compute hypergeometric *p*-values for overrepresentation of each GO term in the specified category among the GO annotations for functions of interest. We selected as true moonlighting function indicators these GO terms with a *p*-value lower than 0.05; this threshold also allows us to remove GO unspecific descriptors (Gomez et al., 2011).

Column 3 of Table 1 shows some examples of identifying moonlighting functions from PPIs. Because the number of interaction partners found in the PPI databases can be high, to pick out the true partners is not an easy task if the researcher has no additional hints. The list of hits has to be properly reduced upon taking into account other bioinformatics predictions as described below or with the help of experimental or clinical data that suggest helpful correlations. In this sense, we have found that, by combining PPI database information and remote homology searches, moonlighting prediction is highly improved. An additional problem is that many species have not been analyzed by interactomics; therefore, a number of MultitaskProtDB proteins have no protein partners in the PPI databases (86 out of 288 proteins of MultitaskProtDB correspond to species without reported experimental interactomics).

In our opinion, the main limit of the level of prediction of multitasking from PPI databases is mainly due to the low sensitivity of interactomics (i.e., many false negatives) rather than low specificity (i.e., false positives).

### Combining interactomics database and Psi-Blast/Bypass searches

We searched for proteins from the MultitaskProtDB database that had interactomics partners in the APID PPI server. As stated above, each moonlighting protein can present a large number of putative PPI partners and also a large output of putative remote homologs from the Psi-Blast algorithm. We have manually inspected both types of output to check whether the intersection of both sets narrows the list of candidate hits and improves the prediction of known moonlighting proteins. This careful manual inspection has been necessary because there is a problem related to the different annotation descriptors depicted by the two types of output. Most Blast/Psi-Blast output from sequence alignments do not report semantic curated annotations, whereas many PPI databases use GO annotations. This fact complicates the automatic matching of the output. We suggest taking as putative positive matches those describing a function in any position of the Psi-Blast/ByPass output that corresponds to a PPI database partner, as shown in the examples from Table 1, columns 3 and 4. We are now designing a program that is able to automatically match two or more outputs. Moreover, the current set of GO annotations contain only about 9500 molecular functions, so most functional annotations from sequence databases cannot be found using GO. Blast2GO annotation (Gotz et al., 2008) should facilitate future analysis, but the current sequence databases contain a lot of ambiguity and low quality descriptors.

Combining the output of interaction partners and remote homologs from PSI-BLAST is the best approach for reducing the sometimes large output from both servers and to improve the bioinformatics prediction of putative moonlighting proteins. Upon overlap Psi-Blast and interaction databases information, as described above, sometimes considering the biological process instead of the accurate molecular function, about half of the moonlighting proteins can be predicted (54 out of 202, as 86 proteins of MultitaskProtDB belong to species which have no interactomics data). Nevertheless, in many cases, the biological process can suggest clues to disclose the moonlighting function.

### Sequence searches using motif/domain programs

**Figure 2:**
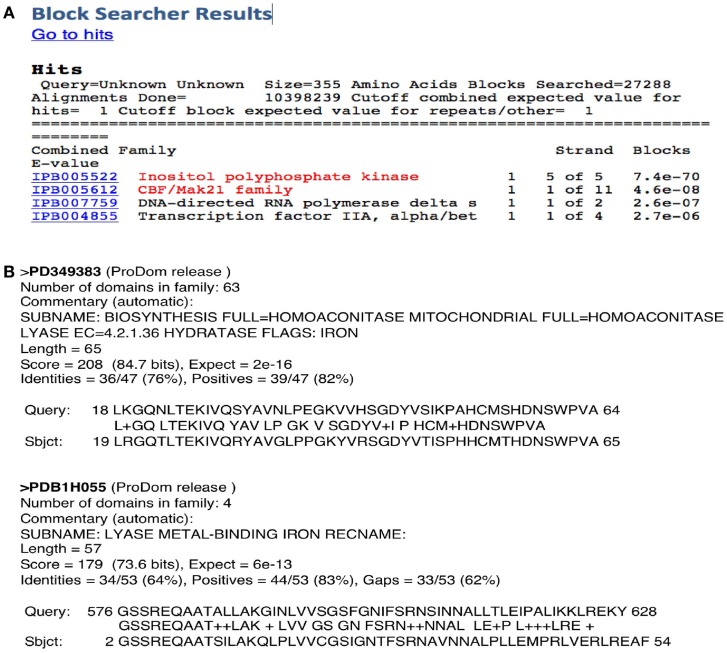
**Two examples of the outputs of two motif/domain programs**. **(A)** Blocks server identifies both functions of the protein Arg 2 in the top positions of the output. **(B)** ProDom program shows two domains related to both canonical and moonlighting functions of aconitase.

Searching for different motifs/domains linked to different function in a target protein sequence using InterPro should, in principle, help to identify moonlighting proteins. However, there are two main problems: (a) the relatively low number of domains and signatures currently known and (b) the current version of programs like Prosite, etc., has been designed for a more accurate prediction of more common motif/domains but does not identify common signatures. This would explain the fact that using InterPro on the MultitaskProtDB proteins discloses the canonical function for about 80% of them, but the moonlighting function in only 8% of cases. For instance, a classical moonlighting example is glyceraldehyde-3-phosphate dehydrogenase (GAPDH)/uracil-DNA-glycosylase (UDG), the Psi-Blast output discloses both functions with high scores, but InterPro only identifies a motif for the canonical function (GAPDH) of this protein. However, both functions are identified by Blocks. In the case of the Arg 2 protein, Blocks identifies the canonical and moonlighting functions as the two top scores of the output (Figure 2A). The fact that pattern detection of a secondary function by a program that has not been updated since 2006 is better than using more modern and refined methods, made us think that this phenomenon may be due to a problem between sensitivity and specificity. Pattern detection tools have been developed traditionally to have a good ratio between specificity and sensitivity. When a gold-standard dataset is built to train these applications, it is usually assumed that all the proteins included in the database have only a unique function. Therefore, if this assumption is not true, as is the case of multifunctional proteins, the program begins biased in terms of loss of sensitivity, so that the tools tend to detect a low number of secondary functions. In this sense, the trend of using much curated seeds of sequences to build these patterns could explain why outdated tools such as Blocks are more effective in detecting secondary functions. If so, this would indicate that to detect such secondary functions, tools such as Blast or Psi-Blast can be more appropriate because they are not dependent on the pre-existence of previously constructed patterns with a limited seed. But this may also be due to other factors, such as the fact that many new tools, in addition to a set of proteins with known function, incorporate a set of false positives (the sequence shares the motif but does not have a lined function). This set contains proteins that contain the pattern associated with the protein function, but do not really perform this function. To check if some of the false positives are actually erroneously discarded for the secondary, moonlighting, functions, we have compared all the set of false positive sequences in the Prosite database with our database of multifunctional proteins. Then, we checked if the patterns corresponding to the sequences of false positives showed a high degree of sequential homology with our multifunctional proteins and if they had a similarity with the secondary function of these multifunctional proteins. This calculation led us to conclude that, at least for Prosite, false positives are true false positives, because none of those functions agreed with the secondary function of the protein.

From the individual programs run together or separately by InterPro, we found and reported previously (Gomez et al., 2003) that ProDom has the best performance at disclosing both canonical and moonlighting domains. This is probably due to the fact that it is a profile database, which is more flexible than pattern search algorithms. It has been generated by an automatic procedure that conserves an important source of variability and, in addition, it has a larger representation of protein families. Figure 2B shows a ProDom prediction of the two domains related to both canonical and moonlighting functions of aconitase. Profile search programs (i.e., Blocks, ProDom) provide a good score, and moreover, the pattern is not only limited to the active site, but it is extended far from the conserved regions. However, they present a poor treatment of the gaps. Pattern search programs like Prosite are more useful for detecting functional active sites but present low specificity, thus slight variations of a pattern will not be detected. The power to identify moonlighting functions by InterPro does not exceed 10%, even adding all the applications included there and considering the correct annotations derived from the supplementary materials present in the descriptors of the patterns. When the putative annotated function corresponds to a new function, the probability of failure in the prediction applying this method is very high, because going from the molecular level to superior ones (cellular, organism, etc.) is risky.

Pfam is, among the programs that InterPro runs, based on Hidden Markov Models (HMM) pattern-search. Pfam families (protein domains clustered using HMM) are built upon multiple sequential alignments, but many of them have been divided into Pfam domains. The biological activity of these families could be described as multiple domains that accomplish together the main function. The limits of these domains are better established than in the family. These features could imply that Pfam domains would be a better tool to identify moonlighting functions. Our results show that Pfam domains are four times more effective in detecting the moonlighting function than other motif and domain methods, but the statistical significance of this difference is low, the *p*-value provided by a X2 test is 0.02.

Another important consideration is that part of the improvement in the prediction of the moonlighting function by Pfam is due to the additional information of the domain function given as supplementary documentation.

Another point that we have explored is the difference between the PfamA and PfamB databases. PfamA is a manually curated database that contains a set of HMMs of more than 14,000 families. The PfamB database is constructed automatically with clusters of sequences produced by the ADDA algorithm (Heger et al., 2005), and their families usually come from alignments that contain proteins with quite heterogeneous functions. This feature encouraged us to test whether PFamB was an appropriate tool to predict secondary functions. We tested both versions using the MultitaskProtDB protein set. PfamA predicts 78% of the canonical functions but only 6% of the moonlighting functions. With PfamB, we found 58 proteins from the MultitaskProtDB protein set that have high homology to at least one PfamB family, and the program properly characterized 60% of the canonical functions and 14% of the moonlighting functions. However, this method is difficult to automate, as the number of annotations to be tested is very high, even selecting the best items previously. In this way, we have made a short list of annotations at every PfamB family, prioritizing longer chain sequences with respect to the short ones included in the original seed sequences used to generate PfamB families. It is also remarkable that about 80% of the multitasking proteins identified by PfamB are not disclosed by PfamA. Obviously, if we have any slight idea of the protein function, the exploration of the PfamB output can provide suggestions on the process of finding the secondary function of our protein.

### Cellular localization of a protein

In a number of cases, moonlighting proteins present each function in a different cellular compartment. Therefore, programs for predicting the cellular localization from protein sequence can help to predict or corroborate a second function. We ran two of these programs: Psort (Nakai and Horton, 1999) and ProtLoc (Cedano et al., 1997). They indicate different predicted localizations in their output according to their respective scores. The two top hits can suggest different tasks for a protein in different localizations. We checked if the two top localization predictions correspond to the canonical and moonlighting functions. Although in some cases they predict correctly the localizations corresponding to both functions, the results are not accurate enough to consider them reliable.

### Mutation correlation analyses

Co-evolution studies of catalytic amino acids, also termed mutation correlation networks, have been used to predict key catalytic residues of enzymes. Anyone who has worked on protein engineering knows that seemingly small changes in the sequence of proteins can sometimes have catastrophic results. The fact that we only publish those mutants that have thrived gives the impression that to obtain more function by mutation is a simple process. Another set of amino acids also contributes to the folding process and to the final functional conformation. Programs of mutation correlation analysis, such as Mistic, can help to identify these amino acids.

We have checked whether the Mistic algorithm can help to predict moonlighting proteins. The main limitation of algorithms like MISTIC is that they require a large multi-alignment; however, current families of moonlighting proteins are scarce, enolases or aldolases being exceptions. In the present work, we analyzed the correlation matrix of the amino acids of enolases that have extra function of binding to plasminogen with the correlation matrix of all the enolases contained in our database. We aligned a set of enolase sequences with <35% of amino acid sequence identity. At the same time, we compared the same set of enolases removing all those that bind plasminogen. We used the first entry in this multiple alignment as a sequence reference in order to facilitate the comparison among both multiple alignments.

**Figure 3:**
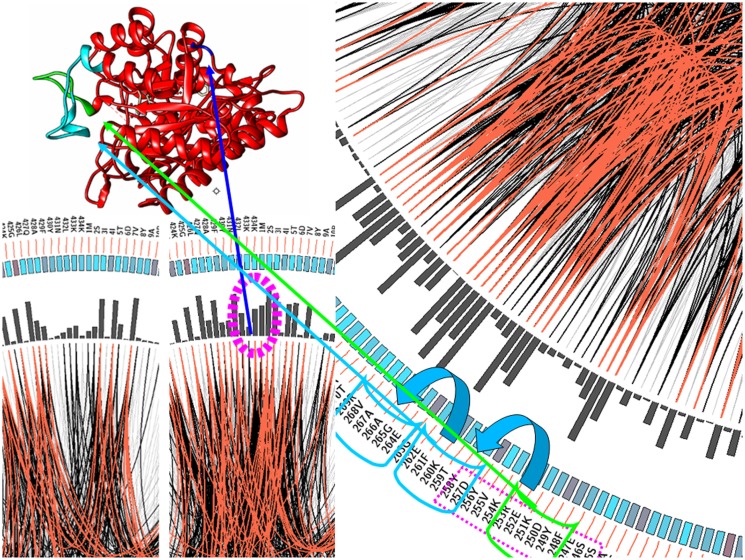
**Enolase mutation correlation analysis**. It can be seen that the areas that have been redesigned to fit the new function of enolase (highlighted in green and navy blue) change the correlation matrix in those regions directly related with the new interaction. However, the modification of a portion of the protein without compromising the network of internal interactions may involve additional changes (depicted here in light blue) in order to maintain the correct conformation and the canonical function of the protein.

As shown in the interactions related to plasminogen binding in Figure 3, the interactions introduced by a new functionality distort the previous net of dependences among the amino acid residues. Still, some distortions are also spread around position 250, another region involved in the interaction. This altered pattern of correlation propagates to position 280. The analysis of the three-dimensional structure of the protein shows that this region is clearly flanking the loop that interacts with plasminogen. That is, the acquisition of new functions seems not to be confined to amino acids normally associated with the binding pattern, but it may involve, in some cases, more global changes in the protein. These observations open up a methodology to find specific positions where there has been a change associated with acquiring a new function.

### Mapping the structural/functional sites on the protein sequence based on sequence homology to 3D structure

There are different algorithms and programs for structure prediction and modeling that can yield hints on functional sites in protein sequences (Pisite, Phyre, I-tasser, SiteEngine…). We have applied two of them: Pisite (Higurashi et al., 2009) and Phyre (Kelley and Sternberg, 2009) to the moonlighting proteins of the MultitaskProtDB database. In the case of Pisite, the main limitation of this program is that it requires that the query protein, or a domain from it, must have a significantly similar amino acid sequence to a structure in the PDB database. Although the amino acid sequence is used in most methods of function prediction, the function of a protein is actually performed by the protein in its native conformation, in other words, by its tertiary or even quaternary structure. It is known that the tertiary structure of a protein is more conserved than the secondary structure and the sequence in relation to the function. For this reason, we believed it would be interesting to see if threading methods, which base their methodology on the recognition of the folding of the protein from the sequence, could help us predict the moonlighting function. To carry out this analysis, we modeled the proteins of our database using Phyre2, considering only the top models with a high degree of confidence and an acceptable percentage of sequence identity. Those models with the same function were combined using only those of the highest percentage of identity. The list of functions obtained from Phyre2 was compared with the canonical and moonlighting functions. For most proteins, the canonical function was identified, but the moonlighting one was identified in only 6% of cases. The rate of correctly identifying moonlighting function is not high, but the results are similar to those obtained by other techniques like Pfam.

**Figure 4:**
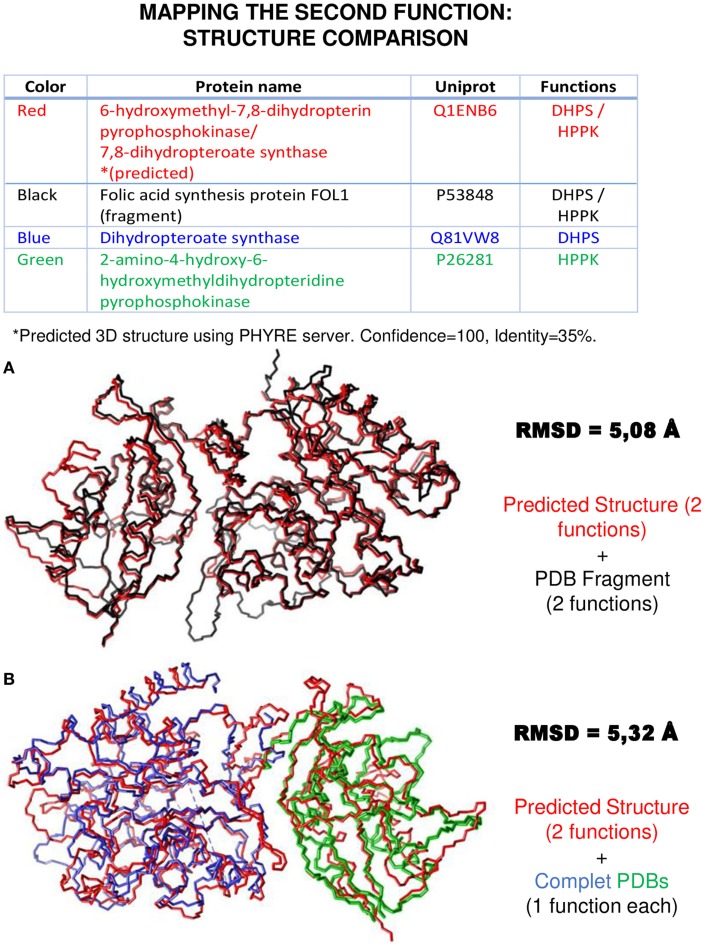
**An example of second function mapping using structural approaches**. The same proteins shown in Figure 1 were used to do a structure comparison using SwissPDBViewer and USCF Chimera. The 3D structure of the “red” moonlighting protein was predicted using Phyre, while the other structures were found in the PDB. In **(A)**, the sequence similarity previously found in Figure 1A was corroborated. In **(B)**, the structure superposition of the three proteins aligned in Figure 1B emphasizes the utility of these methods to map the two functions of a moonlighting protein.

In the case of Pisite, the program identified 266 PDB hits and, in addition to the canonical function, it identifies the moonlighting function for 28 proteins from the MultitaskProtDB. Figure 4A shows an example of a successful match for HPPK and DHPS moonlighting proteins (where Pisite identifies both canonical and moonlighting functions). Moreover, using the SwissPDBViewer tool, both functions can be structurally mapped with a good RMSD (Guex and Peitsch, 1997). PISITE alone cannot identify as many multifunctional proteins as combining Psi-Blast and PPI databases, but it can be used to support putative hits as true positives after running those programs, and interestingly suggest a location on the three-dimensional structure of the moonlighting function. In addition, it can suggest an evolutive origin of the double function as coming from a gene/domain fusion (multitasking) instead of via mutations on a gene (true moonlighting).

Also in the case of Phyre, a table on the top of Figure 4B shows that the program can be used to model and identify the protein folds corresponding to both functions.

### Are multitasking proteins prone to be associated with clinical disorders?

A number of multitasking proteins have been associated with human diseases (Sriram et al., 2005; Jeffery, 2011). In MultitaskProtDB, there are 91 human proteins. A search from the *Online Mendelian Inheritance in Man* (OMIM) database^2^ and from the *Human Gene Mutation Database* (HGMD)^3^ shows that 70% of them are associated with human disorders. This suggests that moonlighting proteins are more prone to be involved in human disease than the average protein. Although the extent of the human proteome is still unknown, it is unlikely that 70% of all proteins are involved in pathologies.

## Discussion

Predicting the function of a protein is a daunting task. It is even more difficult when the protein is multifunctional (Gomez et al., 2003, 2011; Khan and Kihara, 2014; Khan et al., 2014). The prediction of moonlighting proteins could be useful for researchers in tasks like functional annotation of new genomes, and interpreting gene knockout experiments in which deletion out of a gene does not produce the expected results. These types of techniques described above can help to suggest which protein is doing each function, perhaps by means of a moonlighting function, or when interpreting the action and effect of a drug, because it might have an off-target or side effect with somewhat hidden phenotypic traits.

Globally, the remote homology algorithm Psi-Blast had a good performance, but in practice it is difficult for the researcher to pick out the best hits from the large output. As described above, combining different bioinformatics algorithms for protein sequence analysis can help to reduce the number of targets and disclose putative moonlighting proteins. The best approach is to combine protein interactomics databases with Psi-Blast, though at present it still has to be done by manual inspection. This combination leads to the correct prediction of about 50% of the moonlighting proteins, with descriptors that are similar to the desired function, although from a database of proteins previously demonstrated to be multitasking; to perform this task for unknown proteins can be very difficult at present. The proper balance between sensitivity and specificity is especially difficult in the case of the bioinformatics analysis of moonlighting proteins. Kihara’s group have addressed to the problem of finding the remote functions in different ways. The methods, such as the PFP and ESG, have been developed to address remote search of sequences and to work efficiently with GO terms due to their ability to weigh together the GO terms based on their relationship between parent and child terms. The PFP method allows for the diversification of the sequences search by using a non-restrictive *e*-value. On the contrary, the ESG method, instead of doing a unique search, uses the first search to launch new different searches from sequences found in order to expand the explored sequence space in the next step of the search. This fact increases the field of explored sequences. Once these weighted GO term scores result in a single output, Kihara’s group also explore classical tools like PSI-Blast with substitution matrices such as BLOSUM45. This matrix can capture remote homologs in the initial iterations, so this increases the efficiency of the search of moonlighting functions and avoids a PSSM degeneration. Kihara’s group have reduced the number of iterations to only two (Khan et al., 2012, 2014; Khan and Kihara, 2014). In our case, we wanted to use the PSI-Blast algorithm to detect moonlighting functions, but in order to increase the ability to search for remote homologs we used a larger number of iterations, contrary to Kihara’s group. Our strategy is different, but both of them want to increase the sensitivity without compromising the specificity. To illustrate the problem of the specificity and sensitivity in the moonlighting proteins search, we used PfamA and B. These two algorithms represent very well all the methods that are based on profiles or HMM, providing a clear score with which the analyses can be performed. In the case of the PfamA, more restrictive scores are able to find some moonlighting functions with a low number of false positives, but the relaxation of the cutoff increases the proportion of false positives. In contrast to the PfamA protein families, the PfamB profiles are composed of very diverse proteins that represent different functions that, therefore, are much more homogeneous. This functional diversity in the PfamB protein family, and the fact that the sequences included in the PfamB families are not present in the PfamA profiles, increases the sensitivity of the method compared to the PfamA function detection. It has to be taken into account that this increase in the sensitivity has a certain cost in the specificity, because in restrictive cutoff values an acceptable proportion of the true positives compared with the false positives are detected. Even so, when this cutoff parameter is relaxed, the number of false positives suddenly increases about 10 times faster than in the case of the analysis done using PfamA.

The aim of our paper is to explore different methodologies that can be used to search for moonlighting functions. At the same time, we shall try to find possible clues that can make us understand the mechanisms underlying the process of evolution of these moonlighting functions. So, different tools have been explored that would be more or less optimal for this purpose depending on how the protein evolution occurred.

To analyze the moonlighting proteins, different applications have been used; some of them have been previously tested to perform this type of search (Gomez et al., 2003, 2011; Khan et al., 2012, 2014; Khan and Kihara, 2014;, present in the reference list of the manuscript). In our previous works, we have mainly explored the ability of bioinformatics tools to find moonlighting functions more than to calculate the statistical parameters in order to gain insight into the possibility of identifying moonlighting proteins. One of the problems arising from the analysis of these data is how to determine whether there is any correlation between the statistical and the biological significance. It is interesting to determine some statistical parameters to summarize the distribution of the data regarding the biological significance.

As stated by Kihara’s and other groups, the standard method, ROC curves, has a setback, which is the calculation of the number of true negatives. This is not an easy task at all, especially if you are not working with a database designed especially for the purpose of calculating these parameters without problems. In addition, the calculation of a ROC curve is interesting if you are developing a new method in which you want to set a cut-off at which the compromise between specificity and sensitivity is the most appropriate part of the problem that has to be addressed. In our case, we are not yet developing a new method, but rather we are only using the existing methods to show their potential to identify the moonlighting functions. In this sense, it is key to analyze the relationship between true positives and false positives by implementing more restrictive statistical cut-offs. In this way, the noise associated with any classification method is well appreciated because it implies an increase of the false positives when capturing the most remote moonlighting functions in relation to the canonical functions. These parameters give us an idea of the compromise between the sensitivity and the specificity of the method. The analysis of the results facilitates the understanding of what is the range of application of the method. Although all proteins included in the database used have at least one moonlighting function, we could consider that if one was not found it could be considered as a false negative; however, we really do not know if that assumption is in fact true.

When working with PfamA, we do not know if a family with the same function of that profile actually exists in the PfamA database. Although a similar descriptor is present, we cannot be sure that this function really corresponds to the same family of proteins, because it is also possible that this family developed the same function, but using a different structural folding that is not shared with the problem protein. Moreover, false negatives do not exist in the database of profiles; therefore, we could be making a false assumption. In short, we use this type of statistical tool to extract the information from the data we are analyzing, because it does not have the problems associated with the use of the ROC curves.

**Figure 5:**
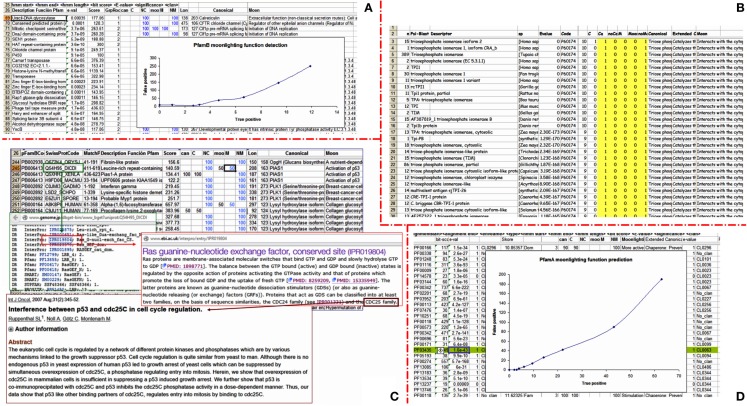
**The problem of moonlighting prediction**. **(A)** The plot shows that databases containing a high heterogeneity of functions, such as PFamB, allow for the identification of non-canonical functions that cannot be found by searching databases of patterns with high functional homology, such as PFamA. However, this implies an increased rate of false positives, when compared with PFamA [as shown in **(D)**]. **(B)** An attempt to exploit the variability of annotation in databases such as a non-redundant database also has its costs, as the hypersaturation of canonical function-annotations contains all sorts of synonyms. **(C)** Checking the reported supplementary documentation can help you to find out relevant details related to the moonlighting function to explore. **(D)** The ratio between false positives and true positives gives us an idea of the compromise between specificity and sensitivity. As we can see when the scores are relaxed, although we are still able to find new moonlighting functions, the number of false positives increases more sharply.

Each method has its own internal procedures, so we have taken some of them to illustrate how the different types of methods work. On one hand, we have used Pfam, which very well illustrates all of the methods based on profiles or HMM, and also provides a clear score with which to perform the analysis (Figure 5). In the case of the moonlighting functions detected by means of PfamA, it appears that with more restrictive scores we are able to find some moonlighting functions including a low number of false positives. Here, the relaxation of the cut-off rapidly increases the proportion of false positives. In this calculation, the canonical functions detected have been removed. The canonical functions identified are much more numerous than the moonlighting and false positives together, but even with the canonical function not being coincident to the moonlighting function, it cannot be considered a false negative because this is describing a real function of the protein. Therefore, in order to simplify the analysis, we have not considered these identified functions as data for our study. The interpretation of these data may tell us that those functions that are remote from the original function will be very difficult to detect by these methods. This is because if we apply more lax criteria to find these remote moonlightings, the number of false positive will grow very quickly, complicating the final analysis of the data. At this point, we must say that, if we had not known the moonlighting function previously, finding some of these moonlighting functions would have been almost impossible. That shows the enormous difficulty that the development of tools to detect such functions will have in the future.

As an example of the methods that employ HMM profiles as the search function of protein families, which usually have highly diverse functions, we used the PfamB (Figure 5B). Interestingly, most of the moonlighting proteins that are detected with this method are not found using the PfamA search. This fact may suggest that we are exploring a different route by means of which moonlighting functions have evolved. It would be interesting, in future studies, to analyze whether the proteins that are detected with PfamB are much more variable in the composition of amino acids of some regions of the protein, because the PfamB profiles are composed of extremely diverse members of proteins with different functions in contrast to the PfamA protein families, which are much more homogeneous.

In the analysis with PfamB, and as a parameter to describe the statistical significance, we have considered the relation between the *e*-value of the PfamB family divided by the score of the members of the PfamB family, which provides the descriptor used for the function. What was observed is that the more restrictive the values, the more the accumulation of a high number of true positives. On the contrary, when this parameter becomes relaxed, the number of false positives rapidly increases, growing about 10 times faster than in the case of the analysis done using PfamA. In other words, in order to get more sensitivity in detecting moonlighting functions with PfamB, we have had to sacrifice a lot of specificity.

For the results obtained using the PSI-Blast, we can see that there is a hyper-saturation of the canonical functions that are, moreover, annotated in the most diverse ways. These descriptors have to be analyzed by concentrating them in a reduced number of expressions, although there are some very abundant descriptors (Figure 5B). There are so many variants in the annotation of the protein function, and this fact complicates the analysis.

The profile-based methods, which take into account larger regions of the protein than pattern-based methods, seem to work better, especially if the sequences used to build the profile have not been excessively refined. This refinement removes so much of the diversity necessary to find the remote members of the functional family of proteins. Other programs, such as those developed by the group of Kihara, have addressed the problem of finding the remote functions in different, specific ways (Khan et al., 2012, 2014; Khan and Kihara, 2014). Methods, such as PFP and ESG, have been developed for remote searches of sequences and for working with GO terms, efficiently, thanks to their ability to jointly weigh GO terms based on their relationship between parent and child terms. PFP allows for a diversification of the search of sequences by using non-restrictive *e*-values. ESG, instead of performing a single search, uses the first search to launch new, different searches from the sequences found in order to expand the explored sequence-space in the next step of the search. This increases the range of explored sequences. Once these weighted scores of GO terms obtained from multiple searches result in a single output, Kihara’s group also explored classical tools, such as PSI-Blast, using substitution matrices such as BLOSUM45. This matrix can capture remote homologs in the initial iterations in order to increase the efficiency of the search for moonlightings functions, and to avoid PSSM degeneration. They have reduced the number of iterations to only two.

In our case, we have also used PSI-Blast to detect moonlighting functions. In order to increase the ability to identify true remote homologs, we have used a larger number of iterations than in Kihara’s work, instead of using the substitution matrix to cover a more distant evolutionary time (high *e*-values; non-restrictive). These are different strategies to increase sensitivity without compromising specificity. In fact, our goal was very different because we really want to exploit the diversity in the source of annotation of the proteins included in the non-redundant databases as a source to obtain the functional annotation rather than design new methods to do the searches.

As previously stated, the aim of this work is not to design new tools for the prediction of the moonlighting functions, but rather to explore whether existing tools can be used to identify multitasking proteins. Some of the approaches used are difficult to systematize or are not easy to be implemented as automatic methods, because they require the interpretation of highly ambiguous and full-of-synonyms written language. Our aim is to explore whether the methods that used hierarchical annotation systems, more appropriate for these purposes, fail. Moreover, assessing the degree of the fitting of the function found with the true moonlighting function is still something extremely subjective, because although it makes it possible to obtain similar results in successive searches, if we search the same query protein running the search against the same database of sequences, the procedure that calculates the similarities is not exempt from some degree of subjectivity. This is inherent in the need of the simplification required to parameterize the descriptors in order to compare them by bioinformatics methods. The different methods to calculate those similarities give us a global idea of similarity, but their scores can vary depending on the method used to score the correct and erroneous predictions. Even if we worked with predefined categories, such as the GO terms, often what they do is to impose constraints in function definition because of the restricted number of GO predefined terms. This causes the loss of some important aspects for protein function although it increases the possibility of a match between the predicted and the original semantic terms. The parent terms in the trees of GO are more unspecific, and these terms will becoming more specific, as long as they are close to the leaves of the GO tree, which is why the identification of the parents functions will have little relevance, in some cases (Figure 5C).

In the GO terms, not all of the information of the function of the protein is condensed. For example, in the case illustrated below, that of the PIAS1 protein, the second function is related to the activation of p53. Well, when we look at the output obtained, the pre-selected subset of functions (detailed in the next paragraph) taken from the descriptors of the proteins included in the seed of PfamB, we see a protein with the same function as the canonical protein, another one with an unknown function, and the Q54H95_DICDI protein that apparently, for the functions described in GO (GO-0005622 intracellular; 0005085 guanyl-nucleotide exchange factor activity; 0051056 regulation of small GTPase mediated signal transduction), keeps a very remote relation to the activation function of p53 activity, as the most similar function. This protein would be related to the regulation of some pathways through a transduction process. Nevertheless, nothing else can be concluded (Figure 5C).

The pre-selected subset of functions extracted from the seed of sequences of the PfamB has been set following the criteria of trying to include proteins that cover long regions that were included in the profile. This was done in order to cover a wide range of profiles and considering that this function has more representatives with the same function descriptors within the PfamB family. Both strategies are trying to avoid those functions that appear in the database just by random coincidence. This strategy obviously will affect the results because we have, for sure, left out some functions that cannot be predicted as moonlighting functions. But, without this screening, the analysis was hardly affordable.

However, if instead of using the GO term we expand the search, we could see that this protein has a common internal standard in a family of proteins (CDC24 and CDC25), which in turn can interact with p53. Now the function we were looking for is closer to the moonlighting function expected and, at the same time, this protein is also involved in regulating the cell cycle. We recognized the correct interaction from among 25,000 proteins of the human genome, and it is also recognized as a regulatory protein, so practically it could give a 100% match. It has to be taken into account that in order to find the correct function, reading the supplementary documentation associated with the profile was necessary. The identification of the real function is not possible only by reading the primary descriptor of the function associated with the GO code. That is why other researchers might not find the correct match, because they may have aborted the search at any time before the localization of the correct reference in the reference list of the paper (Figure 5C).

If we are to make a final conclusion, we could say that the nature of the phenomenon is so varied that it will be difficult to create a single tool to address all the problems of finding the moonlighting functions.

Two additional approaches described previously can help us to consider a protein hit from the methods described as a true moonlighting protein. One of these approaches is the alignment with known 3D structures, which in addition helps to map both functions, on the protein structure and amino acid mutation correlation, which can suggest clues to the evolution of multifunctionality when comparing with mono-functional examples in the family. The second one, the mutation correlation analysis, has been applied to the case of enolase. The structure of enolase has a plasminogen binding site, which is not present in other proteins of the pathogen, although probably the original enolase contained some of the correct amino acids for allowing a strong enough union. In this case, we can see that between five and eight concurrent mutations are needed for the adaptation to occur, but this would eventually involve restructuring other regions of the protein not directly related to the newly acquired function, but maintaining the structure and folding. Enolase has a secondary function that appears quite often in different microorganisms, and this protein allows us to test if the acquisition of a new multifunctionality is a frequently occurring phenomenon or not. If this only occurs very occasionally, the repetition of the same function will be linked to the similarity among the different proteins, indicating that this function has emerged from a common ancestor of the microorganisms containing these enolases. In the opposite case, if none of the proteins share this extra function with any closely related sequence, it could mean that multifunctionality is a frequent event in evolution. The result of these analyses is more consistent with the second hypothesis. This is not a conclusive result, but an interesting clue in the sense that the current list of multifunctional proteins is only a minimal representation of what we can expect.

The type of algorithm that would be needed to detect these moonlighting functions will depend on how we assume that the acquisition of new functions occur. In other words, we could assume that the new features of the protein are produced by small remodeling of the original protein. If so, to detect these small changes will be a complicated task with modern function prediction systems. Because these methods of functional assignment tend increasingly to take into account the global patterns, either of the complete domain or the protein profile, instead of local patterns, like Prosite does. If the moonlighting function is due to a smaller number of amino acids that define the function, the use of a global alignment from a multisequence alignment, either through the use of a matrix (position specific score matrix, PSSM) or a Markov model (HMM), can identify the few amino acids of the moonlighting function on the best global alignment selected by the system. To test whether moonlighting functions are best detected by using predictors of global or local function, we used Pfam, and determined which of the two profiles, A or B types, detected a higher number of moonlighting functions.

Another interesting question is whether moonlighting proteins correspond to network hubs, which, to some extent, can be predicted from network charts. From the set of multitasking proteins used in our previous paper (Gomez et al., 2011), the canonical function of a number of them would correspond to hubs, especially those involved in energy metabolism. This is not a special trove, because it is known that the complexes from interactomics networks with more edges (connections) correspond to energy metabolism and protein synthesis. However, we have not extended this analysis to the full database yet.

At the present state of the art, bioinformatics analysis is better for testing on specific protein cases in which the researcher suspects the possibility of multitasking, from experimental results. Nevertheless, we are currently studying the biology of a model organism, *Mycoplasma genitalium*. This microorganism, a good example of a minimal genome/proteome, is ideal for analyzing and disclosing multitasking proteins. A problem is that more than 20% of the genes/proteins from such a simple microorganism have not been functionally annotated. We have recently reported its proteome at a coverage better than 85% of the predicted genome (Parraga-Nino et al., 2012). We are also re-annotating the proteome for a further bioinformatics prediction of putative moonlighting proteins. However, at present, there are only a few interactions determined experimentally; so, we are working on predictions of protein functions of this microorganism by interactomics. And the bioinformatics methods above are going to be applied.

### Future work

In the present work, we have compared (and matched) coincidences (matches) between different databases or server outputs (Blast/Psi-Blast, PPIs…) manually. This is a time consuming activity and we are trying to design automated tools to do it, incorporating more databases (transcriptomics, knock outs, etc.). But this is not an easy task as the main difficulty is not because of informatics/bioinformatics, it is due to the different annotation criteria used by researchers, usually not using GO terms. In this sense, the manual inspection is more accurate because the researcher can identify relationships not identified by an automated system.

## Author Contributions

Conceived the work: JC, AG, and EQ. Psi-Blast/ByPass and PPI analyses: SH, AG, and AH. Motif/Domain analyses: SH, AC, EQ, and JC. 3D models and mutation correlation analyses: LF, AC, GF, IA, and JC. Drafted and revised the manuscript: EQ, IA, and JC. All authors approved the final version of the manuscript.

## Conflict of Interest Statement

The authors declare that the research was conducted in the absence of any commercial or financial relationships that could be construed as a potential conflict of interest.

## Footnotes

^1^www.geneontology.org

^2^http://www.ncbi.nlm.nih.gov/omim/ or directly entering http://omim.org/

^3^http://www.hgmd.org/
